# What patient reported outcome measures are used in clinical trials in hip fracture? A systematic mapping review

**DOI:** 10.1186/s12891-025-09456-4

**Published:** 2026-01-15

**Authors:** Ruoyu Yin, Zenn Le Hua Soh, Vicky Mengqi Qin, Sara Tasnim, Magdalena Rohr, Christian Apfelbacher, Helen Elizabeth Smith

**Affiliations:** 1 Lee Kong Chian School of Medicine, Nanyang Technological University Singapore, Singapore, Singapore; 2 University Children’s Hospital Regensburg (KUNO), University of Regensburg, Regensburg, Germany; 3 Institute of Social Medicine & Health Systems Research, Otto von Guericke University Magdeburg, Leipziger Street 44, 39120 Magdeburg, Building 2, Office 120, Magdeburg, Germany; 4 School of Medicine, Keele University, Staffordshire, ST5 5BG UK

**Keywords:** Hip fracture, Patient-reported outcome measures, Systematic review, Clinical trials

## Abstract

**Background:**

Patient-reported outcome measures (PROMs) are increasingly used to assess treatment effectiveness in various domains from the patients’ perspective. This systematic review aimed to identify what PROMs have been used in hip fracture clinical trials, whether they are used as the primary outcome, whether validity evidence is referenced and how their use has changed over time.

**Methods:**

Studies obtained from PubMed, Embase, and Web of Science published between 01/01/2010 and 29/09/2025 were assessed. Eligible studies were controlled trials on hip fracture interventions in adult populations published in English. We checked the reference for validity evidence of PROMs used in included studies. Characteristics of each study were extracted, and PROMs usage was summarised by year of publication.

**Results:**

A total of 28 different PROMs were used in 189 trials, with each covering different outcome domains. The most used PROMs were Harris hip score, EuroQoL-5D and pain visual analogue scale. A predominant proportion of studies (*n* = 162, 85.7%) utilised at least one PROMs, including 65 studies used multiple PROMs. There is an increasing trend of PROMs usage in trials and the number of papers using a PROM as a primary outcome over time. However, 95 studies did not reference any validity evidence for PROMs used.

**Conclusion:**

The frequent usage of PROMs in trials, and often as a primary outcome, suggests patient perspective is valued when evaluating hip fracture intervention. However, the lack of a single PROM covering all outcome domains necessitates using more than one PROMs in the included trials. A more comprehensive PROM or a core set of PROMs that measures all patient-related outcomes would achieve a holistic assessment and the ability to make direct comparisons between different interventions.

**Supplementary Information:**

The online version contains supplementary material available at 10.1186/s12891-025-09456-4.

## Introduction

The incidence of hip fracture, a serious injury common in older adults, is increasing along with the aging population, especially in developed countries [1]. By 2050, the global number of hip fractures is predicted to rise to 4.5 million from 1.26 million in 1990 [2]. It can also lead to high cause-specific mortality between 10 and 20% at one year after the injury [3]. Hip fracture has posed a substantial challenge to healthcare because it is often associated with limited mobility and increased vulnerability [4]. Therefore, it is crucial to evaluate the effectiveness of interventions such as hip replacement and arthroplasty on promoting physical functions and enhancing survival in people with hip fractures.

Traditional outcomes for people who had a hip fracture include mortality, readmission to hospital, the length of hospital stays, and the need to further surgery [5]. However, patient-reported outcome measures (PROMs) are increasingly used to assess treatment effectiveness in various domains from the patient’s perspective that are not evaluated by objective examinations or clinical tests [5–7]. PROMs data can also be used to measure the clinical quality of health services, assess the pre-operative health status, and reduce inequalities in healthcare [8]. Additionally, reporting PROMs has the potential to increase provider-patient communication through patient involvement, and may thus enhance patients’ treatment satisfaction [9] and treatment adherence [10, 11]. Examples of PROMs available for hip fracture patients include EuroQol 5-dimension (EQ-5D QoL) [12], Oxford Hip Score (OHS) [13], and Harris Hip Score (HHS) [14].

Despite the benefits offered by PROMs, using PROMs in hip fracture trials is challenging. The lack of consensus on the selection of PROMs and the differences in the domains covered by each PROM result in the inconsistency in PROMs usage in trials [15, 16]. There are also controversies over their variability, reliability, and transparency [17]. Validity and reliability of measures demonstrate their consistent robustness in assessing the intended outcomes [18]. Thus, trials should utilise validated measures and cite the relevant references to guarantee appropriate application of such measures. The use of an unvalidated PROM potentially compromises the quality of the research and diminish its comparability with other studies. Variability in the profile of the target population is another challenge as people with a hip fracture could range from robust to frail and have pre-existing conditions or cognitive impairment [5]. In addition, Core Outcome Measures in Effectiveness Trials (COMET) outlines the essential outcomes, consisting of a comprehensive list of outcomes on mortality/survival, physiological/clinical, life impact, resource use, and adverse effects. As PROMs cover certain COMET domains, identifying which domains were more frequently covered by these PROMs can provide insights into the focus and highlight potential gaps in existing PROMs.

To gain orientation in this field, it is important to gain an overview of the type of PROMs that have been used. As there has been increasing emphasis on patient-centredness in health care over the years, this study aimed to map the PROMs used in hip fracture clinical trials and assess if validity evidence was referenced to support each PROM. While earlier reviews had looked at the variety and validity of these PROMs in total joint arthroplasties [19], it remains unclear whether these trials used PROMs as primary outcomes to further enhance patient-centred research. We have provided a more focused update enhanced by mapping the trends in PROMs usage as a primary outcome specifically in hip fracture trials. This information will enable researchers to better select PROMs for future studies and allow more uniformity across studies for easier comparison of interventions.

### Review questions

In this systematic mapping review, we addressed the following questions:

1. What PROMs are used in hip fracture clinical controlled trials and have they been used as the primary outcome(s)?
2. Which of these PROMs have an evidence-based support for their validity referenced in the respective study?
3. Has the use of PROMs as the primary outcome(s) changed over time?

## Methodology

### Search strategy

Three databases were searched (Embase, PubMed, Web of Science) from 01/01/2010–29/09/2025. We focused on literature published in the recent 15 years to ensure the contemporariness of the review, but also to enable capture of recent trends in this specific research area. Search terms included a combination of (1) hip fracture (e.g. hip fracture, OR intertrochanteric fracture, subtrochanteric fracture, or femoral fracture), OR surgical method (e.g. hip arthroplasty, total hip replacement, or hip hemiarthroplasty), AND (2) controlled trials (e.g., RCT, clinical trials). We have supplemented the literature search with the first 100 results of Google Scholar on 10/10/2022 as a source of grey literature. The threshold of 100 results is based on prior literature, aiming to maintain a balance of a wide scope together with screening efficiency [20–23]. Detailed search strategy in each database is shown in Appendix A. Languages were restricted to English only.

### Eligibility assessment

The de-duplicated library was imported to an artificial intelligence (AI) tool named ASReview, which has been used in other systematic reviews [24–27]. ASReview facilitated screening by actively learning from the human reviewers’ vote of each record and sorting the records according to their relevance. In this way, the records that were more likely to be eligible were prioritised in the list and screened by the human reviewer. Two reviewers (RY and ST) independently screened the titles and abstracts from the literature search on 24/11/2023 and evaluated their eligibility using the AI tool. One reviewer (RY) screened the titles and abstracts from the updated literature search on 29/09/2025. Stopping criteria were predefined as screening the first 1/3 of the total number of records because simulation studies showed the tool’s ability to identify 95% of the eligible studies by screening the first 1/3 of the records [28]. The number of potentially eligible studies was negligible in the remaining 2/3 of the records and thus was excluded by the AI tool. The studies were then imported into Covidence for a full text assessment by two reviewers (RY, ZS, VMQ, ST and MR). Disagreements were resolved through discussion or with a third reviewer (HES or CA).

Studies meeting the following criteria were eligible for inclusion:

4. RCTs/cluster RCTs or controlled trials of hip fracture interventions.
5. Peer-reviewed articles published in English from 01/01/10–29/09/2025 (e.g. full-text articles).
6. Trials whose human participants are patients presenting with hip fracture and receiving acute intervention to manage the fracture itself in a primary, secondary or tertiary healthcare setting.
7. Trials recruiting patients who are adults (above 18 years old).

Studies were excluded if they were:

8. Systematic reviews.
9. Editorials, commentaries, case reports, or letters.
10. Studies based on interventions involving variation of anaesthesia technique or technical details of implantable devices (e.g. radiostereometric studies).
11. Study protocols whose results were already published.
12. Hip fracture studies in the paediatric population (below 18 years old).

### Data extraction

Data extraction was also completed using a predefined data extraction form. We uploaded the full texts of the included studies to SciSpace (https://scispace.com/), an AI data extraction platform, and specified the following study characteristics to extract, including the author, year, setting, country, study design, participant characteristics, sample size, intervention type, PROMs used, whether PROM was the primary outcome, Core Outcome Measures in Effectiveness Trials (COMET) core areas, outcome domains covered by PROM, validity evidence referenced, other outcomes measured and longest follow-up duration. One reviewer checked and corrected the extracted data. The 38-category COMET outcome taxonomy [29] was used to classify the outcome measures used in the PROMs (Appendix B). Appendix C summarises the frequency of COMET core areas and outcome domains covered by the PROMs. The total numbers of each PROM used the studies were summarised by the year of publication. We also calculated the sum of the number of studies that reported PROM(s) as a primary outcome and its percentage of the total number of studies published each year. All outcomes that each study covers were presented according to the COMET taxonomy (Appendix D).

Results were presented following the Preferred Reporting Items for Systematic Reviews and Meta-Analysis (PRISMA) Protocol guidelines with a PRISMA flowchart [30]. Microsoft Excel was used for data analysis and visualisation.

## Results

**Fig. 1:**
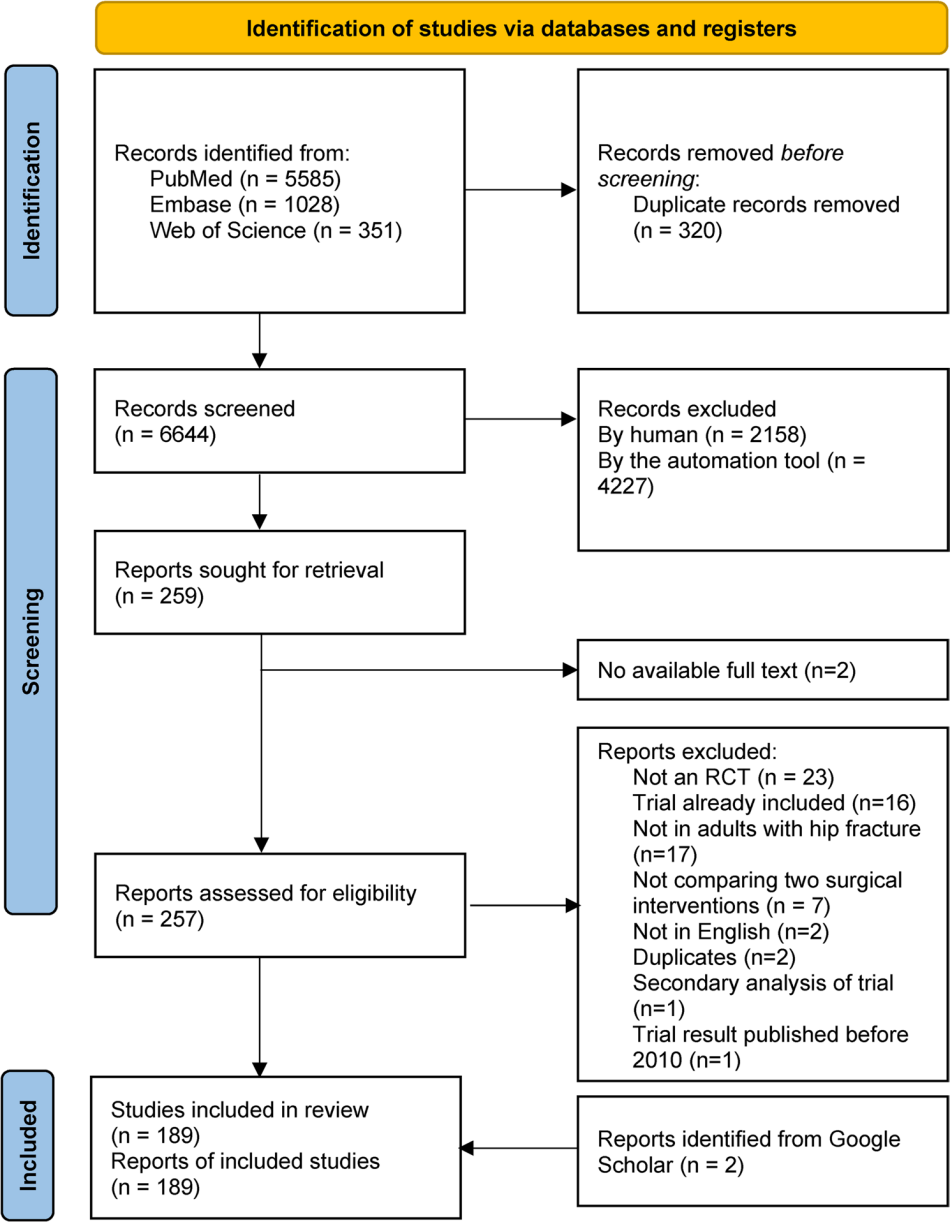
PRISMA flowchart of study selection

As shown in Figs. 1, 6,644 articles were retrieved from the three databases after de-duplication. 2,158 studies were excluded by human reviewers while 4,227 were excluded the AI screening tool. After applying the inclusion and exclusion criteria and full text screening, 189 articles were included [31–219].

Table 1 Shows the characteristics and outcome measurements of each study. The sample sizes ranged from 18 to 2970. Eight studies (4.2%) included in this review are protocols of randomized controlled trials (RCTs) while the rest are completed trials. Studies were predominantly conducted in Asia (*n* = 101), followed by Europe (*n* = 60). 162 studies used one or more PROMs, including 65 studies that used multiple PROMs, while 27 did not use any PROMs. Amongst the studies used PROMs, 37 used the PROM as a primary outcome. Only 52 (32.1%) studies cited the validity for all the PROMs used. A total of 95 studies did not cite any validity evidence for the PROMs used. The remaining 15 studies referenced the validity for some but not all the PROMs used. 

**Table 1:** Characteristics of included studies

Study characteristics	*N* (%)
Total number	189 (100.0%)
Year of publication	
2010–2014	53 (28.0%)
2015–2019	40 (21.2%)
2020–2024	80 (42.3%)
2025	19 (10.1%)
Location	
Asia	101 (53.4%)
Europe	60 (31.7%)
North America	9 (4.8%)
Oceania	7 (3.7%)
Africa	6 (3.2%)
South America	2 (1.1%)
Multiple sites	5 (2.6%)
Sample size (min to max)	18–2970
Study design	
RCT (completed)	171 (90.5%)
RCT (protocol)	8 (4.2%)
Controlled trial	9 (4.8%)
Pilot RCT	1 (0.5%)
Used PROM	
Yes	162 (85.7%)
No	27 (14.3%)
Used PROM as a primary outcome	*N* = 162
Yes	37 (22.8%)
No	25 (15.4%)
NA	100 (61.7%)
Validity evidence for PROM	*N* = 162
Yes	52 (32.1%)
Partial	15 (9.3%)
No	95 (58.6%)

Table 2 shows the 27 PROMs used in the studies, comprising ten generic PROMs, and seventeen specific PROMs focused on the hip, lower limb, or disease, including the Western Ontario and McMaster Universities Arthritis Index (WOMAC) for osteoarthritis. The most commonly used PROM was Harris Hip Score (*n* = 116), the EuroQOL-5D (*n* = 42), and pain VAS (*n* = 35). Sixty-six studies utilised HHS as the sole PROM. The PROMs covered physiological/clinical, and life impact of COMET core areas. PROMs covering the most domains were the SF-36 and MFA. The most frequently covered outcome domain was physical functioning (*n* = 151), followed by musculoskeletal and connective tissue outcomes (*n* = 142) (Appendix C). The least covered COMET outcome domains were cognitive functioning (*n* = 2), global quality of life (*n* = 9), social functioning (*n* = 19) and perceived health status (*n* = 20). Other outcomes measured in the studies (Appendix D) included adverse events (*n* = 180), resource use (*n* = 168), and survival outcomes (*n* = 109).

**Table 2:** Generic & specific proms used and the domains covered by each PROM

Generic PROMs				
Instrument	Number of times the PROM was used (%)	Dimensions/Domains	COMET core area	COMET Outcome Domain
EuroQOL-5D				
Euro Quality Of Life – 5 Dimensions	42 (16.0%)	Mobility	Life impact	25) Physical functioning
		Self-care	Life impact	25) Physical functioning
		Usual activities	Life impact	27) Role functioning
		Pain/discomfort	Physiological/Clinical	9) General outcome
		Anxiety/depression	Life impact	28) Emotional functioning
SF-36				
36-Item Short Form Survey	8 (3.0%)	Physical functioning	Life impact	25) Physical functioning
		Role limitation due to physical functioning or pain	Life impact	27) Role functioning
		Pain (Magnitude)	Physiological/Clinical	9) General Outcome
		Role limitation due to emotional functioning	Life impact	28) Emotional functioning
		General health perceptions	Life impact	31) Perceived health status
		Emotional well-being and vitality	Life impact	28) Emotional functioning
		Social Activities	Life impact	26) Social functioning
		Mental health	Life impact	28) Emotional functioning
SF-36 (PCS)				
36-Item Short Form Survey (Physical Component Score)	1 (0.4%)	Physical functioning	Life impact	25) Physical functioning
		Role limitation due to physical functioning	Life impact	27) Role functioning
		Pain	Physiological/Clinical	9) General Outcome
		General health perceptions	Life impact	31) Perceived health status
SF-12 (PCS)				
12-Item Short Form Survey (Physical Component Score)	6 (2.3%)	Physical functioning	Life impact	25) Physical functioning
		Role limitation due to physical functioning or pain	Life impact	27) Role functioning
		Pain (Magnitude)	Physiological/Clinical	9) General Outcome
		General health perceptions	Life impact	31) Perceived health status
SF-12 (MCS)				
12-Item Short Form Survey (Mental Component Score)	6 (2.3%)	Emotional wellbeing and vitality	Life impact	28) Emotional functioning
		Social Activities	Life impact	26) Social functioning
		Mental health	Life impact	28) Emotional functioning
ICECAP-O				
Investigating Choice Experiments for the Preferences of Older People – CAPability measure for Older people	1 (0.4%)	Social circle and emotional wellbeing	Life impact	28) Emotional functioning
		Independence	Life impact	25) Physical functioning
Pain VAS				
Pain – Visual analogue scale	35 (13.3%)	Pain	Physiological/Clinical	15) Musculoskeletal and connective tissue outcomes
PI-NRS				
Pain Intensity Numeric rating scale	6 (2.3%)	Pain	Physiological/Clinical	15) Musculoskeletal and connective tissue outcomes
Pain				
Pain (yes/no)	7 (2.7%)	Pain	Physiological/Clinical	15) Musculoskeletal and connective tissue outcomes
Energy/fatigue scale				
Energy/fatigue scale	1 (0.4%)	Energy/fatigue	Life impact	28) Emotional functioning 31) Perceived health status
**Specific PROM (Specific to hip**,** lower limb or disease)**				
**Instrument**		**Dimensions/Domains**	**COMET core area**	**COMET Outcome Domain**
WOMAC				
Western Ontario and McMaster Universities Arthritis Index	5 (2.4%)	Pain	Physiological/Clinical	15) Musculoskeletal and connective tissue outcomes
		Stiffness	Physiological/Clinical	15) Musculoskeletal and connective tissue outcomes
		Physical function and mobility	Life impact	25) Physical functioning
OHS				
Oxford hip score	11 (4.2%)	Hip pain	Physiological/Clinical	15) Musculoskeletal and connective tissue outcomes
		Physical function and mobility	Life impact	25) Physical functioning
		Interference with usual work	Life impact	27) Role functioning
HHS				
Harris Hip Score (Composite Measure)	116 (44.1%)	Pain	Physiological/Clinical	15) Musculoskeletal and connective tissue outcomes
		Physical function, walking aids	Life impact	25) Physical functioning
		*Deformity and range of motion (Clinician-reported component*,* NOT patient-reported)*	NA	NA
SMFA				
Short Musculoskeletal Function Assessment	5 (1.9%)	Physical function and mobility	Life impact	25) Physical functioning
		Interference with usual work	Life impact	27) Role Functioning
		Pain in affected limb or back	Physiological/Clinical	15) Musculoskeletal and connective tissue outcomes
		Mental health and emotional well-being	Life impact	28) Emotional functioning
		Interference with social activities	Life impact	26) Social functioning
		Interference with concentration, thinking and memory	Life impact	29) Cognitive functioning
MFA				
Musculoskeletal function assessment	1 (0.4%)	Physical function and mobility	Life impact	25) Physical functioning 31) Perceived health status
		Interference with usual work	Life impact	27) Role Functioning
		Mental health and emotional well-being	Life impact	28) Emotional functioning
		Interference with social activities	Life impact	26) Social functioning
		Interference with concentration, thinking and memory	Life impact	29) Cognitive functioning
LEM				
Lower Extremity Measure	2 (0.8%)	Physical function, mobility and activities of daily living	Life impact	25) Physical functioning
		Interference with social activities	Life impact	26) Social functioning
		Interference with usual day-to-day activities	Life impact	27) Role Functioning
HOS				
Hip outcome Score	2 (0.8%)	Physical function	Physiological/clinical	9) General outcomes
		Interference with mobility	Physiological/clinical	15) Musculoskeletal and connective tissue outcomes
		Interference with mobility & ambulation	Life impact	25) Physical functioning
		Interference with recreational activities	Life impact	30) Global quality of life
		General level of function	Life impact	31) Perceived health status
HSS				
Hospital for special surgery hip rating system	1 (0.4%)	Pain and functional independence	Life impact	25) Physical functioning
		*Muscle power*,* motion and walking (Clinician-reported component*,* NOT patient-reported)*	NA	NA
HOOS				
Hip Disability and Osteoarthritis Outcome Score (HOOS)	1 (0.4%)	Pain	Physiological/Clinical	15) Musculoskeletal and connective tissue outcomes
		Symptoms and stiffness	Physiological/Clinical	15) Musculoskeletal and connective tissue outcomes
		Activities of daily living	Life impact	27) Role Functioning
		Function in sports and recreational activities	Life impact	25) Physical functioning
		Quality of life	Life impact	28) Emotional functioning/well-being 30) Global quality of life
FRS				
Functional Recovery Score	1 (0.4%)	Basic activities of daily living	Life impact	25) Physical functioning
		Instrumental activities of daily living	Life impact	25) Physical functioning
		Mobility	Life impact	25) Physical functioning
NAHS				
Non-Arthritic Hip Score	1 (0.4%)	Pain	Physiological/clinical	15) Musculoskeletal and connective tissue outcomes
		Symptoms	Life impact	25) Physical functioning
		Physical function	Life impact	25) Physical functioning
		Interference with activities	Life impact	25) Physical functioning
JOA hip functional score				
Japanese Orthopaedic Association hip functional score	1 (0.4%)	Pain	Physiological/Clinical	15) Musculoskeletal and connective tissue outcomes
		Range of motion	Life impact	25) Physical functioning
		Ability to walk	Life impact	25) Physical functioning
		Activities of daily living	Life impact	25) Physical functioning
iHOT-12NL				
International Hip Outcome Tool	1 (0.4%)	Pain	Physiological/Clinical	15) Musculoskeletal and connective tissue outcomes
		Health-related quality of life	Life impact	25) Physical functioning
		Physical function	Life impact	25) Physical functioning
		Interference with roles in family	Life impact	27) Role Functioning
		Mental distress	Life impact	28) Emotional functioning/well-being
		Perception of hip problem	Life impact	31) Perceived health status
LEFS				
Lower Extremity Functional Scale	1 (0.4%)	Physical functioning	Life impact	25) Physical functioning
		Social functioning	Life impact	26) Social functioning
UCLA score				
University of California, Los Angeles Score	1 (0.4%)	Physical functioning	Life impact	25) Physical functioning
FJS				
The Forgotten Joint Score	1 (0.4%)	Physical functioning	Life impact	25) Physical functioning
Barthel Index				
Patient-reported Barthel Index	1 (0.4%)	Activities in daily living	Life impact	25) Physical functioning
Total	263 (100.0%)			

**Fig. 2:**
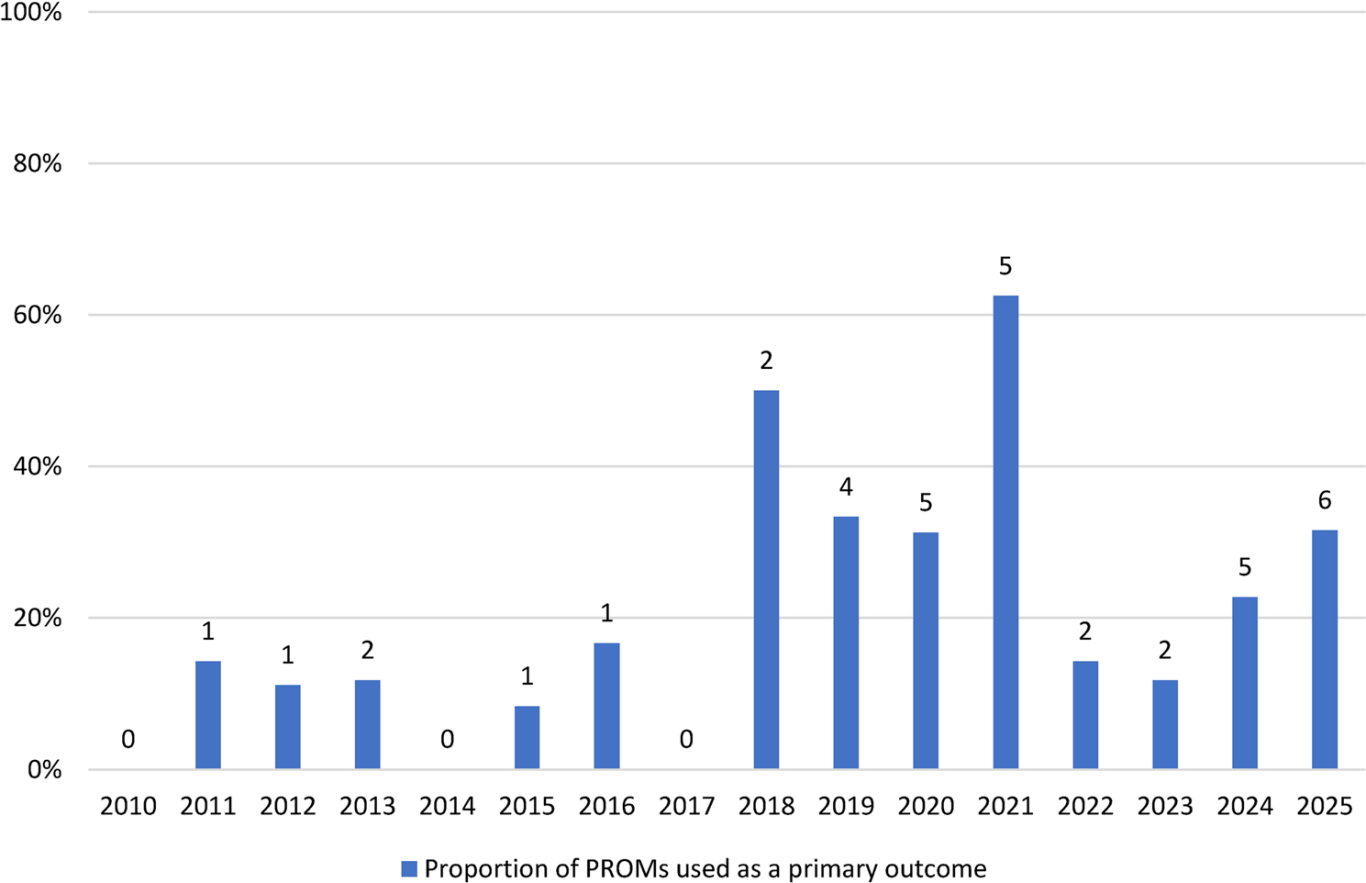
Proportion and number of studies that used patient reported outcome measures (PROM) as a primary outcome over time *The numbers above the bars represent the number of studies that used patient reported outcome measures (PROM) as a primary outcome

Figure 2 shows the use of PROM as a primary outcome in absolute numbers and proportions. Though fluctuating, there was an increasing trend of the proportion of papers that used PROM as a primary outcome over the total number of papers published in that year. In 2025, the number of papers that used PROM as a primary outcome peaked at six, while none of the papers used PROM as a primary outcome in 2010, 2014 and 2017.

## Discussion

Numerous PROMs are employed in hip fracture clinical trials, with each covering different outcome domains. A predominant proportion of studies utilised PROMs, suggesting patient perspectives are valued. More than one-third of the studies used multiple PROMs as no single PROM covered all outcome domains comprehensively. There were fluctuations in PROMs usage in clinical trials and the number of papers using a PROM as a primary outcome over time. In addition, nearly half of the studies failed to reference the validity of the PROMs used.

### Types of PROMs used in hip fracture clinical trials

EQ-5D was the most utilised generic PROM, which is consistent with a previous study that reviewed common hip outcome measures [220]. Although EQ-5D only has five questions, it has good evaluative properties and correlates well to hip-specific PROMs [5] probably because of its comprehensiveness of the domains covered, including mobility, self-care activities, pain/discomfort, and mental health. In addition, it has been included in established core outcome sets for hip fracture as well as national registries such as the National Health System registry in the UK [221]. It can also be used to calculate quality-adjusted life years and subsequent economic evaluation [222]. In contrast, ICECAP-O, an elderly-specific PROM that captures well-being and functional independence, was only used in one study in our review despite hip fracture being a problem most common amongst the elderly. Accessibility of the tool should not be a barrier; it has been translated into 15 languages besides English and is free to use. However, despite being developed over 10 years ago, awareness of ICECAP-O, in orthopaedic research is poor, unlike in economic and social care research [16].

The most frequently used hip-specific PROM was the HHS, a composite measure comprising both patient-reported and clinician-reported outcomes. This is consistent with a previous review that reported PROM usage in total hip arthroplasty [19]. Since its development in 1969 as one of the first outcome measures for hip surgery [223], HHS has continued to be widely used. It is available without cost, can be administered by a health professional without training and takes only five minutes to complete, factors that alongside familiarity continue to support its use [224]. Whilst a popular measure of hip function, HHS cannot be considered a pure PROM because it can be reported by patients or clinicians. Its limitations include clinician bias and ceiling effect, which is the inability of a rating tool to properly evaluate the patient’s ability if the highest score is achieved, thus affecting its effectiveness and validity [225, 226]. Nevertheless, modified HHS is a pure PROM, as it removed the clinical evaluation domain from HHS, i.e., range of motion and deformity [227]. Albeit useful for short-term follow-ups, the responsiveness of HHS decreased after 5 years, compared with SF-36 [228]. Therefore, it is recommended that studies with long-term follow-ups incorporate other PROMs as outcomes as well. Surprisingly, the OHS, a PROM specific to total hip replacements, was rarely used, though also included in the NHS PROM registry in UK [221]. A previous systematic review recommended combining OHS and WOMAC for hip joint evaluation as they are easy to complete and not susceptible to clinician bias [220], but this combination was not used in the included studies.

### COMET domains covered by PROMs

In addition, a wide range of PROMs were used in the included studies, as no single PROM covered all the COMET domains. The most addressed COMET outcome domains were physical functioning and musculoskeletal outcomes, not surprising as gait and mobility are hip-dependent. Less frequently covered domains included social functioning, perceived health status and cognitive functioning. This raises concern because mobility affects social activity and interaction [229, 230], which in turn causes depressive states and reduced cognitive functioning [231, 232]. This deficit in cognition could undermine the reliability of PROMs. Although COMET serves as a taxonomy rather than a checklist, the gaps in the less frequently covered domains highlight current shortcomings and indicate the need for a more comprehensive PROM or a core set of PROMs to enable holistic assessment and strengthen future research to maximise patient benefit.

### PROM usage over time

Encouragingly, PROMs were used in most trials, suggesting the acknowledgement of value of patient perspective in research. There was an increasing trend of the proportion of studies that used PROMs as a primary outcome over the total number of papers published in that year over the years, which is consistent with another systematic review on ankle fracture interventions that demonstrated a promising proportion of PROM as the primary outcome measure [233]. We also found fluctuating number of PROMs used over time, and overall, there was an increasing trend in the proportion. The pattern we observed was not as clear as that observed when focusing on PROMs usage in hip and knee arthroplasty [19] in four major journals [19]. Our review of publications was much broader (searching all journals indexed in the three academic databases) and more focused (hip fracture management only). In addition, we included other surgical methods besides hip arthroplasty. All factors may explain the less optimistic finding of our review of PROM usage.

### Lack of reporting of validity in trials

Despite the popularity of PROMs in the included studies, a small proportion of studies did not use any PROMs or did not provide a citation of validity evidence for the PROMs utilised in their trial. This may suggest lack of confidence in using PROMs and lack of awareness of the need to ensure the validity of the measures used. To enhance the rigour of PROM use requires at a research organisational level adequate training, administrative support and ensuring sufficient resources [234, 235]. Funding bodies could also play a part ensuring the proposed PROMs are justified, with supporting evidence of validity and reliability. Using PROMs with validity evidence will increase the trustworthiness of the results and aid in the comparative analysis of patient outcomes and satisfaction after hip fractures.

### Barriers to PROM implementation

Barriers to implementing PROMs may deter usage in hip fracture trials. PROMs usually involve questionnaires that require users to have higher cognitive functions like memory and judgement [236]. Therefore, the high prevalence of cognitive impairment in patients with hip fracture (47% [4]) makes PROMs usage difficult. Eleven studies included in our review excluded cognitively impaired patients, which could reduce the generalisability of their findings. In 2024, a Delphi study on assessing health-related related quality of life in people with cognitive impairments recommended the combination of proxy-report, self-report and clinician observation. Such an approach could minimise the issue and improve inclusiveness of the participants in PROMs [237]. Blinding of patients, doctors and researchers are necessary to avoid reporting and detection biases [4]. Objective measurements of physical functions, imaging and biomarkers are recommended to accompany PROMs when blinding is impossible [6, 16]. Repeated measurements may be used to reduce the variability of PROMs and provide evidence on the disease progression and process of recovery over time [238–240].

### Recommendations for policy, research and practice

The findings of this review have implications for those working in many areas including policy, research, education and clinical practice, as improving the quality and patient centredness of healthcare is a multidisciplinary endeavour that needs to be proactive. Health care service developers need to be attentive to the needs of patients in parallel with the needs of the health professionals. Funding agencies should insist on the inclusion of PROMs all funded trials. Educationalists must consider how best to promote teaching about PROMs in training programs, and when needed access to resources that can provide practical guidance on the use of PROMs in both clinical practice and research. Further research is needed to expand the current recommended core outcome set of five domains (mortality, pain, activities of daily living, mobility, and health-related quality of life) [200] for hip fracture trials. Work should also aim to develop PROMs to cover those underrepresented but relevant domains for older people, including social functioning, perceived health status and cognitive functioning.

### Strengths and limitations

This review is a valuable update of PROMs used in hip fracture studies and reviews the trends in their usage. However, it has several limitations. First, only three databases and one language were reviewed due to time and resource constraints. We have adopted a novel AI-based title and abstract screening tool to facilitate the screening. Two thirds of the records were excluded by the automation tool, so it is possible that potentially eligible studies were missed. However, the validity of the tool was demonstrated by simulation studies [28] and two reviewers independently used the tool to screen the titles and abstracts and compared the results. Second, classification of the COMET outcome domains covered by each PROM is subjective because some questionnaires had vague questions that may be categorised differently. For example, the EQ-5D asks about usual activities which could be classified as both role functioning and physical functioning. To mitigate the potential source of bias, one reviewer (RY or ZS) classified the outcomes according to the COMET taxonomy, and a second reviewer (CA, HES, or VMQ) checked all classifications. Any discordance was discussed until a consensus was reached. Third, most studies were conducted in the Asian and European populations while few studies involved African or Latin American populations; considering the differences in cultural contexts, the current findings may not be generalisable to these less represented countries. Lastly, we did not search for eligible studies in the excluded systematic reviews at the titles and abstract screening stage because of restricted manpower, this may result in missing a potentially eligible study.Additionally, one of the 189 included studies was retracted after screening [91]. Although it was retained solely to describe outcome usage, its reporting should be interpreted with caution, and its inclusion may slightly affect the frequency of reported outcomes.

## Conclusion

In conclusion, many different PROMs are being utilised across hip fracture clinical trials, with each covering different outcome domains. We found a high overall proportion of included studies using PROMs, but many studies did not provide validity reference of these PROMs. The proportion of studies using PROMs as primary outcome over the total number of papers published in that year fluctuated, with few using PROMs as primary outcome. The fact that most studies have incorporated PROMs suggests patient perspective is valued when evaluating hip fracture intervention. However, the lack of a single PROM covering all outcome domains necessitates using more than one PROMs in the included trials. A more comprehensive PROM or a core set of PROMs with established validity that measures all patient-related outcomes would provide a holistic assessment and the ability to make direct comparisons between different interventions. Future research is recommended to utilise PROMs with validity and reliability as primary outcomes and cover other outcome domains to fully capture patient experience and improve quality of healthcare.

## Supplementary Information

Supplementary Material 1.

## Data Availability

All data generated or analysed during this study are included in this published article and its supplementary information files.

## Abbreviations

AI: Artificial intelligence
COMET: Core Outcome Measures in Effectiveness Trials
EuroQoL-5D: EuroQol 5-dimension
HHS: Harris Hip Score
ICECAP-O: ICEpop CAPability measure for Older people
iHOT-12NL: Dutch version of International Hip Outcome Tool 12-item
OHS: Oxford Hip Score
PROM: Patient-reported outcome measures
PRISMA: Preferred Reporting Items for Systematic Reviews and Meta-Analysis
