# Supplementary material for: What patient reported outcome measures are used in clinical trials in hip fracture? A systematic mapping review
Source: BMC Musculoskelet Disord. 2026 Jan 15;27:74. doi: 10.1186/s12891-025-09456-4 (PMC12853937; doi:10.1186/s12891-025-09456-4)
Supplement: Supplementary file 1 — Supplementary Material 1. [file 12891_2025_9456_MOESM1_ESM.docx]

## **Appendix A: Literature search strategy**

PubMed

(trial[Title/Abstract] OR "Controlled Clinical Trial"[pt] OR "Clinical Trial"[pt] OR (Controlled Clinical Trial[MeSH Terms]) OR randomised[Title/Abstract] OR randomised[Title/Abstract])

AND

(("arthroplasty replacement hip"[Title/Abstract] OR "hip replacement arthroplastie*"[Title/Abstract] OR "hip replacement arthroplasty"[Title/Abstract] OR "total hip arthroplasty"[Title/Abstract] OR "hip prosthesis implantation*"[Title/Abstract] OR "total hip replacement*"[Title/Abstract] OR ("hip fracture*"[Title/Abstract] OR "intertrochanteric fracture*"[Title/Abstract] OR "subtrochanteric fracture*"[Title/Abstract] OR "trochanteric fracture*"[Title/Abstract] OR "femoral fracture*"[Title/Abstract] OR "femoral neck fracture*"[Title/Abstract] OR "hemiarthroplasty"[Title/Abstract] OR "hip injuries"[Title/Abstract])))

Limit Published date to: 20100101- 20231124

Limit language to: English

Embase

1. hip fracture/ or hip injury/ or joint fracture/ or femoral head fracture/ or femoral neck fracture/ or femur intertrochanteric fracture/ or femur pertrochanteric fracture/ or femur subtrochanteric fracture/ or femur trochanteric fracture/
2. total hip prosthesis/ or hip prosthesis/ or hip replacement/ or total hip replacement.mp.
3. total hip replacement/ or total hip arthroplasty/ or hip hemiarthroplasty/ or hip arthroplasty/
4. 1 or 2 or 3
5. Limit Published date to: 20100101-20231124
6. Limit language to: English
7. Limit species to: Human
8. Limited age to: Adults (18-64 years old) or Aged (65 years old and above)
9. Limit articles to: Randomised controlled trials and controlled clinical trial

Web of Science

1. TS=(Hip fracture* OR hip injury OR joint fracture* OR femoral head fracture* OR femoral neck fracture* OR femoral intertrochanteric fracture* OR femur pertrochanteric fracture* OR femur subtrochanteric fracture* OR femur trochanteric fracture* OR total hip prosthesis OR hip prosthesis OR hip hemiarthroplasty OR hip replacement OR total hip replacement) NOT TS=(Child* OR teen* OR youth* OR infant* OR neonate* or p*ediatric*) NOT TS=(total knee replacement or knee replacement or knee or upper extremity)
2. TS=(adult* or elderly or geriatric*)
3. TS=(Randomi*ed controlled trial* OR RCT* OR Controlled clinical trial*) AND TS=(Treatment outcome*) NOT TS=(systematic review* or meta-analysis)
4. 1 AND 2 AND 3
5. Limit Published date to: 20100101-20231124
6. Limit language to: English
7. Limit species to: Human
8. Limited age to: Adults (18-64 years old) or Aged (65 years old and above)

## **Appendix B: 38-category Core Outcome Measures in Effectiveness Trials (COMET) outcome taxonomy**

| **Core Area** | Mortality/ survival | Physiological/clinical | Life impact | Resource use | Adverse events/ effects |
| --- | --- | --- | --- | --- | --- |
| **Outcome Domain** | 1) Mortality/ survival | 2) Blood and lymphatic system outcomes | 25) Physical functioning | 34) Economic | 38) Adverse events/ effects |
|  |  | 3) Cardiac outcomes | 26) Social functioning | 35) Hospital |  |
|  |  | 4) Congenital, familial and genetic outcomes | 27) Role functioning | 36) Need for further intervention |  |
|  |  | 5) Endocrine outcomes | 28) Emotional functioning/ well-being | 37) Societal/ carer burden |  |
|  |  | 6) Ear and labyrinth outcomes | 29) Cognitive functioning |  |  |
|  |  | 7) Eye outcomes | 30) Global quality of life |  |  |
|  |  | 8) Gastrointestinal outcomes | 31) Perceived health status |  |  |
|  |  | 9) General outcomes | 32) Delivery of care |  |  |
|  |  | 10) Hepatobiliary outcomes | 33) Personal circumstances |  |  |
|  |  | 11) Immune system outcomes |  |  |  |
|  |  | 12) Infection and infestation outcomes |  |  |  |
|  |  | 13) Injury and poisoning outcomes |  |  |  |
|  |  | 14) Metabolism and nutrition outcomes |  |  |  |
|  |  | 15) Musculoskeletal and connective tissue outcomes |  |  |  |
|  |  | 16) Outcomes relating to neoplasms: benign, malignant and unspecified (including cysts and polyps) |  |  |  |
|  |  | 17) Nervous system outcomes |  |  |  |
|  |  | 18) Pregnancy, puerperium, and perinatal outcomes |  |  |  |
|  |  | 19) Renal and urinary outcomes |  |  |  |
|  |  | 20) Reproductive system and breast outcomes |  |  |  |
|  |  | 21) Psychiatric outcomes |  |  |  |
|  |  | 22) Respiratory, thoracic and mediastinal outcomes |  |  |  |
|  |  | 23) Skin and subcutaneous tissue outcomes |  |  |  |
|  |  | 24) Vascular outcomes |  |  |  |

## **Appendix C: COMET outcome domains covered the patient reported outcome measures**

| Core Outcome Measures in Effectiveness Trials (COMET) | Number of times the outcome domain was covered (%) |
| --- | --- |
| **Total ^1^** | 493 (100.0%) |
| **Physiological/clinical** |  |
| 9) General outcome | 46 (11.6%) |
| 15) Musculoskeletal and connective tissue outcomes | 142 (35.9%) |
| **Life impact** |  |
| 25) Physical functioning | 151 (38.1%) |
| 26) Social functioning | 19 (4.8%) |
| 27) Role functioning | 54 (13.6%) |
| 28) Emotional functioning | 50 (12.6%) |
| 29) Cognitive functioning | 2 (0.5%) |
| 30) Global quality of life | 9 (2.3%) |
| 31) Perceived health status | 20 (5.1%) |

**^1^** All PROMs covered more than one domain, leading to a total count of 493.

## **Appendix D: All COMET outcomes domains covered in each study**

| **Reference** | **PROM usage** | **Adverse events** | **Resource use** | **Clinical outcomes** | **Life impact** | **Survival outcomes** |
| --- | --- | --- | --- | --- | --- | --- |
| Adeel 2020 | Yes | Yes | Yes | Yes | Yes | No |
| Agrawal 2017 | Yes | Yes | Yes | Yes | Yes | Yes |
| Aktselis 2013 | Yes | Yes | Yes | Yes | Yes | Yes |
| Andalib 2020 | Yes | Yes | Yes | Yes | Yes | No |
| Andreani 2015 | Yes | Yes | Yes | Yes | Yes | Yes |
| Auffarth 2011 | Yes | Yes | Yes | Yes | Yes | Yes |
| Bajpai 2015 | Yes | Yes | Yes | Yes | Yes | No |
| Ball 2024 | Yes | Yes | Yes | Yes | Yes | Yes |
| Bartels 2022 | Yes | Yes | Yes | Yes | Yes | Yes |
| Barwar 2014 | No | Yes | Yes | Yes | Yes | No |
| Bhalodia 2025 | Yes | Yes | Yes | Yes | Yes | No |
| Bhandari 2019 | Yes | Yes | Yes | Yes | Yes | Yes |
| Boonyanuwat 2025 | Yes | Yes | Yes | Yes | Yes | No |
| Borges 2020 | No | Yes | Yes | Yes | Yes | Yes |
| Bretherton 2016 | No | Yes | Yes | Yes | Yes | No |
| Cao 2014 | Yes | Yes | Yes | Yes | Yes | Yes |
| Cha 2025 | Yes | Yes | No | Yes | Yes | No |
| Chammout 2016 | Yes | Yes | Yes | Yes | Yes | Yes |
| Chandrashekhar 2024 | Yes | Yes | Yes | Yes | Yes | No |
| Changbao 2024 | Yes | Yes | Yes | Yes | Yes | No |
| Chatterji 2022 | Yes | Yes | Yes | No | Yes | No |
| Chaudhary 2024 | Yes | Yes | Yes | Yes | Yes | Yes |
| Chechik 2014 | Yes | Yes | Yes | Yes | Yes | Yes |
| Cheng 2014 | Yes | Yes | Yes | Yes | Yes | Yes |
| Clement 2021 | Yes | Yes | No | Yes | Yes | Yes |
| Deangelis 2012 | Yes | Yes | Yes | No | Yes | Yes |
| Desteli 2015 | Yes | No | Yes | Yes | Yes | No |
| Dhamangaonkar 2013 | Yes | Yes | Yes | Yes | Yes | No |
| Dragosloveanu 2022 | Yes | Yes | Yes | Yes | Yes | Yes |
| El 2016 | Yes | Yes | Yes | Yes | Yes | Yes |
| Emami 2013 | Yes | Yes | No | Yes | Yes | Yes |
| Farey 2022 | No | Yes | Yes | Yes | Yes | Yes |
| Fernandez 2022 | Yes | Yes | Yes | Yes | Yes | Yes |
| Figved 2018 | Yes | No | Yes | Yes | Yes | Yes |
| Fitzpatrick 2011 | No | No | Yes | Yes | No | No |
| Gavaskar 2014 | Yes | Yes | Yes | Yes | Yes | Yes |
| Griffin 2013 | Yes | Yes | Yes | Yes | Yes | Yes |
| Griffin 2014 | Yes | Yes | Yes | Yes | Yes | Yes |
| Griffin 2019 | Yes | Yes | Yes | Yes | Yes | Yes |
| Griffin 2021 | Yes | Yes | Yes | Yes | Yes | Yes |
| Guerra 2014 | Yes | No | No | No | Yes | Yes |
| Guo 2013 | Yes | Yes | Yes | Yes | Yes | Yes |
| Haddon 2019 | No | Yes | Yes | Yes | Yes | Yes |
| Hamdy 2025 | Yes | Yes | Yes | Yes | Yes | No |
| Haq 2014 | Yes | Yes | Yes | Yes | Yes | No |
| Harshwardhan 2024 | Yes | Yes | Yes | Yes | Yes | No |
| Hazowary 2025 | Yes | Yes | Yes | Yes | Yes | No |
| Hedbeck 2011 | Yes | Yes | Yes | Yes | Yes | Yes |
| Hedbeck 2013 | Yes | Yes | Yes | Yes | Yes | Yes |
| Hemant 2024 | Yes | Yes | Yes | Yes | Yes | No |
| Hempel 2024 | Yes | Yes | Yes | Yes | Yes | Yes |
| Hopp 2016 | Yes | Yes | Yes | Yes | Yes | Yes |
| Hoseth 2025 | Yes | Yes | No | Yes | Yes | Yes |
| Huang 2017 | Yes | Yes | Yes | Yes | Yes | No |
| Iorio 2019 | No | Yes | Yes | No | No | Yes |
| Jain 2022 | Yes | Yes | Yes | Yes | Yes | No |
| Javdan 2013 | Yes | Yes | No | Yes | Yes | No |
| Jeffcote 2010 | Yes | Yes | Yes | Yes | Yes | Yes |
| Jianbo 2019 | Yes | Yes | Yes | Yes | Yes | No |
| Kalland 2019 | Yes | Yes | Yes | Yes | Yes | Yes |
| Kalsbeek 2020 | Yes | Yes | Yes | Yes | Yes | No |
| Kassem 2022 | Yes | Yes | Yes | Yes | Yes | Yes |
| Kazemian 2013 | Yes | Yes | Yes | Yes | Yes | Yes |
| Kazemian 2016 | Yes | Yes | Yes | Yes | Yes | Yes |
| Khan 2021 | Yes | Yes | Yes | Yes | Yes | Yes |
| Kos 2011 | Yes | Yes | Yes | No | Yes | Yes |
| Kouvidis 2012 | Yes | Yes | Yes | Yes | Yes | Yes |
| Kumar 2015 | Yes | Yes | No | Yes | Yes | No |
| Kumar 2019 | Yes | Yes | Yes | Yes | Yes | Yes |
| Kumar 2023 | Yes | Yes | Yes | Yes | Yes | Yes |
| Kumar 2024 a | No | Yes | Yes | Yes | Yes | No |
| Kumar 2024 b | Yes | Yes | Yes | Yes | Yes | No |
| Langlois 2015 | No | Yes | Yes | Yes | Yes | Yes |
| Langslet 2014 | Yes | Yes | Yes | Yes | Yes | Yes |
| Liang 2022 | Yes | Yes | Yes | Yes | Yes | Yes |
| Lim 2020 | Yes | Yes | No | Yes | Yes | Yes |
| Lin 2022 | Yes | Yes | Yes | Yes | Yes | No |
| Liu 2015 | Yes | Yes | Yes | Yes | Yes | No |
| Makeen 2021 | Yes | Yes | No | Yes | Yes | Yes |
| Makridis 2010 | No | Yes | Yes | Yes | Yes | Yes |
| Malhotra 2025 | Yes | Yes | Yes | Yes | Yes | No |
| Manson 2022 | Yes | Yes | Yes | No | Yes | Yes |
| Martí-Garín 2023 | No | Yes | Yes | No | No | Yes |
| Matre 2013 | Yes | Yes | Yes | Yes | Yes | Yes |
| Mayank 2023 | Yes | Yes | Yes | No | Yes | Yes |
| McCormack | Yes | Yes | Yes | Yes | Yes | Yes |
| Mehta 2024 | Yes | Yes | Yes | Yes | Yes | No |
| Mitsuzawa 2023 | No | Yes | Yes | Yes | Yes | No |
| Mittal 2017 | Yes | Yes | No | Yes | Yes | No |
| Moradi 2021 | Yes | Yes | Yes | Yes | Yes | Yes |
| Movrin 2020 | Yes | Yes | Yes | No | Yes | Yes |
| Mural 2024 | Yes | Yes | Yes | Yes | Yes | No |
| Ni 2022 | Yes | Yes | Yes | Yes | Yes | No |
| Nie 2024 | Yes | Yes | Yes | Yes | Yes | No |
| Noree 2025 | Yes | Yes | Yes | Yes | Yes | Yes |
| Okcu 2013 | Yes | Yes | Yes | Yes | Yes | Yes |
| Okcu 2015 | Yes | Yes | Yes | Yes | Yes | No |
| OlarteSalazar 2023 | Yes | Yes | No | Yes | Yes | Yes |
| Ono 2024 | No | Yes | Yes | Yes | Yes | No |
| Othman 2025 | Yes | Yes | Yes | Yes | Yes | No |
| Panagopoulos 2023 | Yes | Yes | Yes | Yes | Yes | No |
| Park 2010 | No | Yes | Yes | Yes | Yes | No |
| Parker 2010 | No | Yes | Yes | Yes | Yes | Yes |
| Parker 2010 | No | Yes | Yes | Yes | Yes | Yes |
| Parker 2012 | Yes | Yes | Yes | Yes | Yes | Yes |
| Parker 2015 | No | Yes | Yes | Yes | Yes | Yes |
| Parker 2017 | No | Yes | Yes | Yes | Yes | Yes |
| Parker 2019 | Yes | Yes | Yes | Yes | Yes | Yes |
| Parker 2020 | No | Yes | Yes | Yes | Yes | Yes |
| Parker 2020 | No | Yes | Yes | Yes | Yes | Yes |
| Parker 2025 | Yes | Yes | Yes | Yes | Yes | Yes |
| Paswan 2024 | No | Yes | No | Yes | Yes | Yes |
| Patterson 2021 | Yes | Yes | Yes | Yes | Yes | Yes |
| Peng 2020 | Yes | Yes | Yes | Yes | Yes | No |
| Prabhat 2025 | Yes | Yes | Yes | Yes | Yes | No |
| Prakash 2022 | Yes | Yes | Yes | Yes | Yes | No |
| Rashed 2021 | Yes | Yes | No | Yes | Yes | Yes |
| Reindl 2015 | Yes | Yes | No | Yes | Yes | Yes |
| Renken 2012 | Yes | Yes | No | Yes | Yes | Yes |
| Sadeghpour 2024 | Yes | Yes | Yes | Yes | Yes | No |
| Saglam 2025 | Yes | Yes | Yes | Yes | Yes | Yes |
| Saikia 2023 | Yes | Yes | Yes | Yes | Yes | No |
| Sarkar a 2024 | Yes | Yes | Yes | Yes | Yes | No |
| Sarkar b 2024 | Yes | Yes | Yes | Yes | Yes | No |
| Schemitsch 2023 | Yes | Yes | Yes | Yes | Yes | Yes |
| Selim 2020 | Yes | Yes | Yes | Yes | Yes | No |
| Shankar 2025 | Yes | Yes | Yes | Yes | Yes | No |
| Shannon 2019 | Yes | Yes | Yes | Yes | Yes | No |
| Sharma 2016 | Yes | Yes | Yes | Yes | Yes | Yes |
| Shen 2023 | Yes | Yes | Yes | Yes | Yes | No |
| Shin 2017 | Yes | Yes | Yes | Yes | Yes | No |
| Shyamdhar 2023 | Yes | No | No | Yes | Yes | No |
| Siavashi 2015 | Yes | Yes | Yes | Yes | Yes | No |
| Singh 2017 | Yes | Yes | Yes | Yes | Yes | Yes |
| Singh 2019 | Yes | Yes | Yes | Yes | Yes | Yes |
| Singh 2025 | Yes | Yes | Yes | Yes | Yes | No |
| Sivakumar 2024 | Yes | Yes | Yes | Yes | Yes | Yes |
| Slobogean 2021 | Yes | No | No | No | Yes | No |
| Sonaje 2018 | Yes | Yes | Yes | Yes | Yes | No |
| Sten 2014 | Yes | Yes | Yes | Yes | Yes | Yes |
| Stern 2011 | No | Yes | Yes | Yes | Yes | Yes |
| Stoffel 2013 | Yes | Yes | No | Yes | Yes | No |
| Talsnes 2013 | No | Yes | Yes | No | No | Yes |
| Tao 2013 | Yes | Yes | Yes | Yes | Yes | Yes |
| Taylor 2012 | Yes | Yes | Yes | Yes | Yes | Yes |
| Tellefsen 2023 | No | Yes | Yes | Yes | No | No |
| Thanchuingam 2023 | Yes | Yes | Yes | Yes | Yes | No |
| Thuppad 2025 | Yes | Yes | Yes | Yes | Yes | No |
| Tilaveridis 2023 | No | Yes | Yes | Yes | Yes | Yes |
| Tol 2024 | Yes | Yes | Yes | Yes | Yes | Yes |
| Ugland 2018 | Yes | Yes | Yes | Yes | Yes | Yes |
| Uikey 2024 | Yes | Yes | Yes | No | Yes | No |
| Upadhayay 2023 | Yes | Yes | Yes | Yes | Yes | Yes |
| Vaquero 2012 | Yes | Yes | Yes | Yes | Yes | Yes |
| Vekris 2011 | Yes | Yes | Yes | Yes | Yes | Yes |
| Verettas 2010 | Yes | Yes | Yes | Yes | Yes | No |
| Verzellotti 2020 | Yes | Yes | Yes | Yes | Yes | Yes |
| Viberg 2020 | Yes | Yes | Yes | Yes | Yes | Yes |
| Vidovic 2013 | Yes | Yes | Yes | Yes | Yes | Yes |
| Wadekar 2025 | Yes | Yes | Yes | Yes | Yes | No |
| Wang 2018 | Yes | Yes | No | Yes | Yes | No |
| Wang 2019 | No | No | Yes | Yes | No | No |
| Wang 2023 | Yes | Yes | Yes | Yes | Yes | No |
| Wang 2023 | Yes | Yes | Yes | Yes | Yes | Yes |
| Watson 2013 | Yes | Yes | Yes | Yes | Yes | Yes |
| Wei 2020 | Yes | Yes | Yes | Yes | Yes | Yes |
| Wen 2025 | Yes | Yes | Yes | Yes | Yes | No |
| WinnockdeGrave 2012 | No | Yes | Yes | Yes | Yes | Yes |
| Wolf 2020 | Yes | Yes | Yes | Yes | Yes | Yes |
| Wolf 2020 | Yes | Yes | Yes | No | Yes | Yes |
| Woolnough 2025 | Yes | Yes | Yes | Yes | Yes | Yes |
| Wu 2014 | Yes | Yes | Yes | Yes | Yes | Yes |
| Xu 2010 | Yes | Yes | Yes | Yes | Yes | Yes |
| Xu 2010 | Yes | Yes | Yes | Yes | Yes | No |
| Yamauchi 2014 | Yes | Yes | Yes | Yes | Yes | No |
| Yang 2011 | Yes | Yes | Yes | Yes | Yes | Yes |
| Yeo 2025 | Yes | Yes | Yes | Yes | Yes | Yes |
| Yin 2024 | Yes | Yes | Yes | Yes | Yes | No |
| Zehir 2015 | Yes | Yes | Yes | Yes | Yes | Yes |
| Zhang 2013 | Yes | Yes | Yes | Yes | Yes | Yes |
| Zhang 2019 | Yes | Yes | Yes | Yes | Yes | No |
| Zhang 2022 | Yes | Yes | Yes | Yes | Yes | No |
| Zhang 2023 | Yes | Yes | Yes | Yes | Yes | No |
| Zhao 2020 | Yes | No | No | Yes | Yes | No |
| Zhao 2022 | Yes | Yes | Yes | Yes | Yes | Yes |
| Zhou 2012 | Yes | Yes | Yes | Yes | Yes | No |
| Zhu 2012 | Yes | Yes | Yes | Yes | Yes | No |
| Zkayan 2015 | Yes | Yes | Yes | Yes | Yes | No |
| Zügner 2024 | Yes | No | No | Yes | Yes | No |
